# Rural–urban health disparities for mood disorders and obesity in a midwestern community

**DOI:** 10.1017/cts.2020.27

**Published:** 2020-03-24

**Authors:** Christi A. Patten, Young J. Juhn, Euijung Ryu, Chung-Il Wi, Katherine S. King, Josh T. Bublitz, Robert J. Pignolo

**Affiliations:** 1 Department of Psychiatry and Psychology, Mayo Clinic, Rochester, MN, USA; 2 Department of Pediatric and Adolescent Medicine, Mayo Clinic, Rochester, MN, USA; 3 Division of Biostatistics and Informatics, Mayo Clinic, Rochester, MN, USA; 4 Internal Medicine, Mayo Clinic, Rochester, MN, USA

**Keywords:** Disparities, mood disorders, obesity, geography, socioeconomic status

## Abstract

**Introduction:**

Prior studies indicate greater disease burden for obesity among rural compared with urban residents but no differences for mood disorder based on geographic location. Recent attention has focused on the need to examine regional rural–urban disparities in disease burden. We focused on mood disorders and obesity prevalence within three southeastern Minnesota counties served by the Mayo Clinic Center for Translational Science Award, in Rochester, Minnesota, as these were top priorities identified in community health needs assessments.

**Methods:**

Cross-sectional study to assess the association of rural–urban locality on 5-year (2009–2014) prevalence of mood disorder and obesity obtained using the Rochester Epidemiological Project medical records linkage system, among subjects residing in three mixed rural–urban counties on April 1, 2014. Multivariable analyses adjusted for demographics, socioeconomic status using an individual housing-based measure, and counties.

**Results:**

The study cohort (percent rural location) included 91,202 (15%) for Olmsted, 10,197 (51%) in Dodge, and 10,184 (57%) in Wabasha counties. On multivariate analysis, 5-year prevalence of mood disorders and obesity was significantly greater for urban compared with rural residents, after adjusting for confounders; odds ratios (95% confidence intervals): 1.21 (1.17–1.26), *P* < 0.001, and 1.05 (1.01–1.10), *P* = 0.016, respectively. Observed effects were not modified in additional models adjusted for health care utilization (HCU; ≥1 general medical examination visit and flu vaccination).

**Conclusions:**

Rural–urban health disparities for burden of mood disorders and obesity are independent of socioeconomic status and HCU in a Midwestern community. It is important to assess potential regional heterogeneity of rural–urban disparities on health outcomes.

## Introduction

About 14% of the US population lives in rural areas [1]. Research has generally found that rural populations face major health disparities compared with urban regions [2]. For example, residents of rural counties in the USA are more likely to have poorer health outcomes along a variety of domains of health quality, including health behavior, morbidity factors, clinical care, and physical environment features [3].

In this study, we examined rural–urban health disparities in the prevalence of obesity and any mood disorder diagnosis over a 5-year period, in three local Minnesota counties served by our National Institutes of Health (NIH)-funded Clinical and Translational Science Award (CTSA), with different proportions of residents living in rural areas. We focused on these two health outcomes, because mental health and obesity were identified as the top two community health priorities from 2014 to 2016 in all three counties [4–7]. In addition, a survey involving a convenience sample of 418 community members statewide in Minnesota (10% rural residence) revealed that mental health and wellness (e.g., obesity and physical activity) were reported as top health needs [8]. Consistent with a bi-directional approach to community engagement [9], we seek to understand the influence of rural–urban geographic location on disparities in health outcomes identified as important to our local communities.

Large nationally representative studies indicate that individuals residing in rural locations have greater prevalence of obesity than their urban counterparts, after adjusting for individual-level socioeconomic status (SES) measures, for example, income and education [3,10–14]. Less attention has focused on *place characteristics* that may explain disparities in obesity, including neighborhood or housing features. Wen *et al.* [15] found that the effect of rurality on obesity was minimal when accounting for neighborhood-level features (e.g., walkability). There is limited research on geographic health disparities for mood disorders, but interestingly, epidemiological studies observed no differences between rural and urban residents [16–18].

Recently, research and policy attention has turned to population-based, preventive approaches for improving the physical, mental, and social well-being of rural residents [19]. The current literature on rural health disparities is largely derived from national surveillance data which may not reflect regional nuances. A recent white paper of the National Academy of Medicine suggested empowering people by delivering clinical care in their personal and social context as one of the four action priorities for vital directions of the US healthcare system [20]. In this context, assessing and addressing rural–urban health disparities at a local or community level is conceptually and logistically important for CTSA hubs serving their local populations. For example, in a predominantly rural region, Hill *et al.* [21] found that severity of obesity was worse among Black compared to White persons and for *urban compared to rural* residents. These types of regional data provide important baseline information for local county health departments and healthcare systems to measure the success of community-engaged mental and behavioral health promotion efforts over time.

In this study, we hypothesized a higher prevalence of obesity and mood disorder for rural compared to urban residents within each Minnesota county studied. Based on social determinants of health framework [22–24], we build on the current literature on rural–urban health disparities in disease burden by accounting for (1) health care utilization (HCU) [25] and (2) HOUSES, a unique, individual-level, composite, and objective SES index, derived from individual housing-based features [26].

## Materials and Methods

The study was approved by the Institutional Review Boards of Mayo Clinic and Olmsted County Medical Center.

### Setting and Populations

**Fig. 1.**
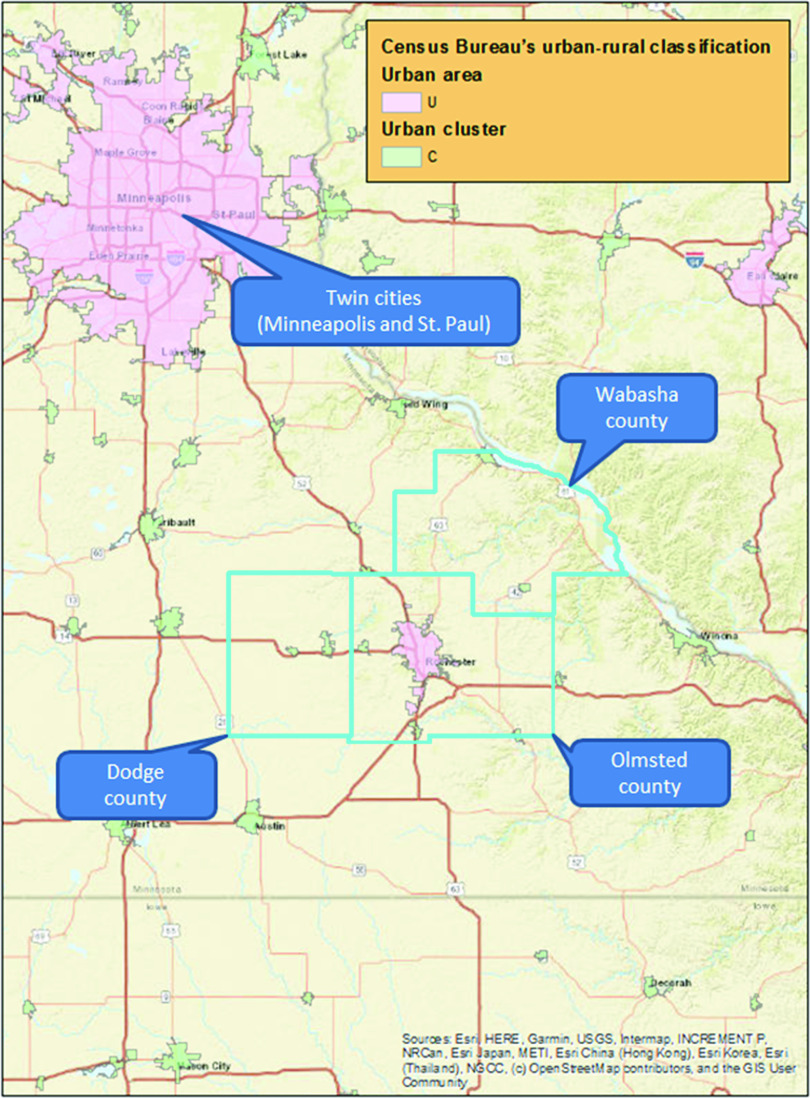
Study setting and urban–rural classification for Olmsted, Dodge, and Wabasha southeastern Minnesota counties*.

*Note*: *There are two indicators for urban area (pink) and urban cluster (light green). We combined urban areas and urban cluster into “Urban” classification for this study. “Rural” encompasses all population, housing, and territory not included within “Urban”.

The study included three southeastern Minnesota counties: Olmsted, Dodge, and Wabasha. Fig. 1 illustrates the study setting, including urban–rural classification. Based on US Census Bureau and the American Community Survey data [27], the proportion of rural residents is estimated at 16.5%, 51.8%, and 64.5% in Olmsted, Dodge, and Wabasha, respectively. The Rochester Epidemiology Project (REP) [27] links data on medical care delivered to the populations of Olmsted, Dodge, and Wabasha counties in Minnesota. The majority of medical care in these communities is provided by the Mayo Clinic and Olmsted Medical Center in Rochester, along with Mayo Health System sites located in Kasson and Wabasha. The health care records from these institutions are linked together through the REP records linkage system. Medical records for nearly all residents are available for clinical research. The health records available through the REP capture a high proportion of the population in these three counties (99.9% in Olmsted, 89.0% Dodge, and 87.0% in Wabasha counties) [27]. In this study, we used the REP census to identify all individuals who resided in each county on April 1, 2014. We excluded those who had refused research authorization in all three counties. In 2014, 90.9% of eligible patients provided research authorization (91.3% among men and 90.6% among women) [27].

### Study Design

We performed a cross-sectional study using a population-based design to assess the association of rurality with 5-year prevalence (between April 1, 2009 and March 31, 2014) of any mood disorder and obesity diagnoses within each of three Minnesota counties, using the REP medical records linkage system.

### Study Subjects

The patient cohort period for each county was formed with those who (1) were 18 years of age or older; (2) had authorized use of their medical records for research; (3) had individual-level SES measure defined by the HOUSES index and developed and validated by the study team (see Measures below), available; and (4) had rurality status available.

### Measures

#### Demographics

Study subject characteristics assessed were age (on April 1, 2014), sex, and race/ethnicity (non-Hispanic Whites versus others).

#### Rural–urban geographic location

Rurality status was defined by the US Census Bureau’s [28] rural–urban classification. The Census Bureau’s urban areas represent densely developed territory and encompass residential, commercial, and other non-residential urban land uses. The Census Bureau delineates urban areas after each decennial census by applying specified criteria to decennial census and other data. Briefly, the US Census Bureau uses criteria including total population thresholds, density, land use, and distance, and Census blocks are the “building blocks” for urban areas [29]. We used shape profiles provided by the US Census Bureau for rural–urban classification to specifically join the study subjects’ geocoded addresses. The Census Bureau identifies two types of urban areas: urbanized areas (UAs) of 50,000 or more people and urban clusters (UCs) of at least 2500 and less than 50,000 people. “Rural” encompasses all population, housing, and territory not included within an urban area. We combined UAs and UCs into “Urban” classification for this study.

#### Socioeconomic status

Our study team developed an innovative SES measure called HOUSES, overcoming the absence of individual-level objective SES measures in data sources frequently used for clinical and epidemiological studies and avoiding use of aggregate-level SES measures as a proxy for individual SES due to its significant misclassification bias [30,31]. For generating the HOUSES index [26], addresses for study subjects were retrieved from the REP [27], which collects and maintains all historical individual addresses during residence in each of the three counties, and matched against to real property data publicly available. The HOUSES index is a robust individual measure of SES represented by a single factor made up of four housing features: number of bedrooms, number of bathrooms, square footage of the unit, and estimated building value of the unit, ascertained from the county Assessor’s office [26]. It is a standardized index score by summing the z-scores for each housing variable (i.e., standardized index). The higher the HOUSES (z-score and quartile converted from z-score), the higher the SES. The HOUSES index makes a unique contribution to the measurement of SES beyond income and education [32]. Construct validity was also found such that HOUSES index predicts a broad range of health behavior (e.g., smoking exposure) and outcomes (e.g., obesity, preterm birth, and general health) conceptually and empirically known to be associated with SES.

#### Health care utilization

HCU was defined as having at least one clinic visit for general medical examination (GME) during the study period. We also assessed if subjects had at least one Current Procedural Terminology code for flu vaccination during the study period.

#### Mood disorders and obesity diagnoses

We assessed prevalence of any mood disorder (major depressive disorder, dysthymia, bipolar disorder I, bipolar disorder II, and anxiety disorder) and obesity diagnoses. The diagnostic indices of the REP [33] were searched electronically to extract the International Classification of Diseases, Ninth Revision (ICD-9) codes of these two diagnoses in the medical records of the patients within each county that were ever assigned by any health care institution from April 1, 2009 through March 31, 2014 (i.e., 5-year prevalence with a single ICD code). These ICD-9 codes were grouped into clinical classification codes proposed by the AHRQ-Healthcare Cost and Utilization Project.

### Statistical Methods

Count (percentage) was used to describe categorical data, while median (1st quartile – 3rd quartile) was used to describe continuous data. Chi-square tests were used to assess the differences between rural and urban characteristics within each county of interest. For each county, the relationships of rural–urban locality with mood disorder and obesity were assessed with logistic regression models adjusting for age, sex, race/ethnicity (non-Hispanic White vs. others), and SES (HOUSES). Additional combined analysis was also conducted by adding counties as an adjusting factor to estimate an overall association result. HCU metrics were included in additional multivariable logistic models (separate models for at least 1 GME clinic visit and flu vaccination, respectively) to adjust for potential impact of HCU on the association between rural–urban locality and mood disorder and obesity. As secondary analysis, we conducted age-stratified analysis (18–45, 46–65, and >65) to determine if associations between rural–urban locality and each outcome were similar by age group. We also conducted a multivariable analysis for mood disorder, adjusting for obesity. R statistical software, version 3.42 (Vienna, Austria), and SAS software package, version 9.4M05 (SAS Institute Cary, NC, USA), were used for analysis. Level of significance was set at 0.05 for all tests.

## Results

### Characteristics of Study Subjects

The study cohort consisted of 91,202 residents from Olmsted, 10,197 from Dodge, and 10,184 from Wabasha counties. Slightly over half of subjects were women in each county (Table 1). The percentage of non-White subjects was 18.3% in Olmsted, 8.6% in Dodge, and 7.1% in Wabasha counties. Based on the HOUSES index, Olmsted had a higher proportion of subjects in the lowest SES quartile (25.8%) compared to Dodge (23.8%) or Wabasha (13.9%) counties. HCU based on GME visit was 65.7% (Olmsted), 61.4% (Dodge), and 57.5% (Wabasha). For flu vaccine, the respective percentages were 68.9%, 67.9%, and 64.1%. The proportion of subjects with rural residence was 15.1% in Olmsted, 51.1% in Dodge, and 60.0% in Wabasha counties. Supplementary Figure S1 shows density of HOUSES SES rural–urban status in all three counties.

**Table 1.** Study subject characteristics by county

Characteristic	Olmsted (*N* = 91,202)	Dodge (*N* = 10,197)	Wabasha (*N* = 10,184)
Age (median (25th–75th %tile))	46 (31.76–60.22)	48 (33.59–60.62)	52 (35.22–65.25)
Female (*N*(%))	48,691 (53.4%)	5305 (52.0%)	5301 (52.1%)
Race/Ethnicity (*N*(%))			
African American	4446 (4.9%)	37 (0.4%)	36 (0.4%)
Asian	4767 (5.2%)	50 (0.5%)	41 (0.4%)
Hispanic	4638 (5.1%)	565 (5.5%)	354 (3.5%)
Non-Hispanic White	74,471 (81.7%)	9319 (91.4%)	9459 (92.9%)
Other/Unknown	2880 (3.2%)	226 (2.2%)	294 (2.9%)
Socioeconomic status (HOUSES) quartiles (*N* (%))			
Q1	23,495 (25.8%)	2430 (23.8%)	1413 (13.9%)
Q2	23,315 (25.6%)	2394 (23.5%)	2435 (23.9%)
Q3	22,777 (25.0%)	2580 (25.3%)	2991 (29.4%)
Q4	21,615 (23.7%)	2793 (27.4%)	3345 (32.9%)
General medical examination visit (*N*(%))	59,856 (65.7%)	6252 (61.4%)	5850 (57.5%)
Flu vaccination (*N*(%))	62,778 (68.9%)	6911 (67.9%)	6530 (64.1%)
Rural locality (*N*(%))	13,853 (15.2%)	5208 (51.1%)	5803 (57.0%)
Any mood disorder (*N*(%))	26,257 (28.8%)	2755 (27.0%)	2557 (25.1%)
Obesity (*N*(%))	16,273 (17.8%)	2133 (20.9%)	1614 (15.9%)

Prevalence of mood disorder was 28.8% (Olmsted), 27.0% (Dodge), and 25.1% (Wabasha). Obesity prevalence was 17.8% (Olmsted), 20.9% (Dodge), and 15.8% (Wabasha).

### Univariate Associations of Rural–Urban Locality and Subject Characteristics

Table 2 displays the findings on the univariate associations of locality (rural vs. urban) and subject characteristics within each of the three counties. Across each of the three counties, compared to rural residents, those residing in *urban areas* were significantly younger and were more likely to be women, minorities, and with low SES. Moreover, the proportion of subjects with at least one preventive care clinic visit and flu vaccination was lower for urban residents compared to rural in Olmsted and Wabasha counties, while the proportion was similar for Dodge county study subjects.

**Table 2.** Univariate associations of rural–urban locality and subject characteristics by county

	Olmsted county			Dodge county			Wabasha county		
	Rural (*N* = 13,853)	Urban (*N* = 77,349)	*P* value	Rural (*N* = 5208)	Urban (*N* = 4989)	*P* value	Rural (*N* = 5803)	Urban (*N* = 4381)	*P* value
Age			<0.001			<0.001			<0.001
18–45	5660 (40.9%)	39,275 (50.8%)		2177 (41.8%)	2653 (53.2%)		2172 (37.4%)	1905 (43.5%)	
46–65	5908 (42.6%)	25,039 (32.4%)		2067 (39.7%)	1530 (30.7%)		2284 (39.4%)	1405 (32.1%)	
>65	2285 (16.5%)	13,035 (16.9%)		964 (18.5%)	806 (16.2%)		1347 (23.2%)	1071 (24.4%)	
Female	6979 (50.4%)	41,712 (53.9%)	<0.001	2650 (50.9%)	2655 (53.2%)	0.018	2954 (50.9%)	2347 (53.6%)	0.008
Race/ethnicity			<0.001			<0.001			<0.001
African American	87 (0.6%)	4359 (5.6%)		17 (0.3%)	20 (0.4%)		16 (0.3%)	20 (0.5%)	
Asian	200 (1.4%)	4567 (5.9%)		19 (0.4%)	31 (0.6%)		15 (0.3%)	26 (0.6%)	
Hispanic	504 (3.6%)	4134 (5.3%)		239 (4.6%)	326 (6.5%)		154 (2.7%)	200 (4.6%)	
Non-Hispanic White	12,757 (92.1%)	61,714 (79.8%)		4818 (92.5%)	4501 (90.2%)		5454 (94.0%)	4005 (91.4%)	
Other/Unknown	305 (2.2%)	2575 (3.3%)		115 (2.2%)	111 (2.2%)		164 (2.8%)	130 (3.0%)	
HOUSES quartiles			<0.001			<0.001			<0.001
Q1	1418 (10.2%)	22,077 (28.5%)		715 (13.7%)	1715 (34.4%)		600 (10.3%)	813 (18.6%)	
Q2	2872 (20.7%)	20,443 (26.4%)		970 (18.6%)	1424 (28.5%)		1294 (22.3%)	1141 (26.0%)	
Q3	3740 (27.0%)	19,037 (24.6%)		1448 (27.8%)	1132 (22.7%)		1754 (30.2%)	1237 (28.2%)	
Q4	5823 (42.0%)	15,792 (20.4%)		2075 (39.8%)	718 (14.4%)		2155 (37.1%)	1190 (27.2%)	
General medical examination visit	9422 (68.0%)	50,434 (65.2%)	<0.001	3211 (61.8%)	3041 (61.1%)	0.477	3536 (61.0%)	2314 (52.8%)	<0.001
Flu vaccination	10,003 (72.2%)	52,775 (68.3%)	<0.001	3506 (67.4%)	3405 (68.4%)	0.305	3912 (67.4%)	2618 (59.8%)	<0.001
Mood disorder	3450 (24.9%)	22,807 (29.5%)	<0.001	1189 (22.8%)	1566 (31.4%)	<0.001	1357 (23.4%)	1200 (27.4%)	<0.001
Obesity	2310 (16.7%)	13,963 (18.1%)	<0.001	1016 (19.5%)	1117 (22.4%)	<0.001	915 (15.8%)	699 (16.0%)	0.798

Prevalence of mood disorder was significantly *greater for urban* as compared with rural residents in Olmsted (29.5% vs. 24.9%), Dodge (31.4% vs. 22.8%), and Wabasha (27.4% vs. 23.4%) counties (all *P* < 0.001). Obesity prevalence was significantly higher among urban than rural residents in Olmsted (18.1% vs. 16.7%, *P* < 0.001) and in Dodge (22.4% vs. 19.5%, *P* < 0.001) counties. However, in Wabasha county, obesity prevalence was similar for urban and rural study subjects (16.0% vs. 15.8%, *P* = 0.798).

### Multivariable Association of Rural–Urban Locality with Prevalence of Mood Disorder and Obesity

Table 3 presents the multivariable associations of rural–urban locality with mood disorder and obesity prevalence by county and combined counties. After adjusting for age group, sex, race/ethnicity, and SES confounders, the association of *urban residence and increased likelihood* of mood disorder remained statistically significant for each county. Odds ratios (OR) and 95% confidence intervals (CIs) were 1.19 (1.14–1.24) for Olmsted, 1.37 (1.24–1.50) in Dodge, and 1.16 (1.06–1.27) for Wabasha counties. The combined analysis showed that prevalence of mood disorders was higher for urban compared with rural residents (OR = 1.21, 95% CI: 1.17–1.26; *P* < 0.001).

**Table 3.** Multivariable associations of rural–urban locality with mood disorder and obesity by county and combined counties*^,+,#^

	Odds ratio	Lower 95% confidence limit	Upper 95% confidence limit	*P* value
Olmsted				
Mood disorder	1.19	1.14	1.24	<0.001
Obesity	1.04	0.99	1.10	0.088
Dodge				
Mood disorder	1.37	1.24	1.50	<0.001
Obesity	1.14	1.02	1.26	0.016
Wabasha				
Mood disorder	1.16	1.06	1.27	0.002
Obesity	1.01	0.91	1.13	0.821
Combined counties				
Mood disorder	1.21	1.17	1.26	<0.001
Obesity	1.05	1.01	1.10	0.016

* All analyses examined the effect of rural–urban location (Reference = Rural) on the condition of interest. Analyses were adjusted for age group (in 2014), sex, race/ethnicity (non-Hispanic White vs. other), socioeconomic status (HOUSES) quartiles.

+ Two additional multivariate models included HCU (1 or more general medical examination visits, and flu vaccination, respectively) which did not modify the observed effects.

# In the analysis for combined county, the multivariable model added counties as additional adjusting factors.

After adjusting for covariates, the effect of urban residence on obesity was attenuated for Olmsted county (OR = 1.04, CI: 0.99–1.10; *P* = 0.088), attenuated but remained significant in Dodge county (OR = 1.14, CI: 1.02–1.26; *P* = 0.016), and remained non-significant for Wabasha county (OR = 1.01, CI: 0.91–1.13; *P* = 0.821) (Table 2). The combined analysis found that overall prevalence of obesity was *greater for urban residents* compared with rural residents (OR = 1.05, 95% CI: 1.01–1.10; *P* = 0.016).

Additional multivariable analyses were conducted that adjusted for any GME visit and flu vaccination, respectively. Results remained unchanged when adding these additional variables to the models for rural–urban locality effects on mood disorder and obesity prevalence. For mood disorders, we also adjusted for obesity prevalence in the above models but found no impact on the observed association with urban locality.

We conducted age-stratified analyses to further explore the observed urban effect on prevalence of mood disorders and obesity. Results indicate that for both conditions, the strongest associations are observed in the middle-age group (46–65 years) compared with those 18–45 or >65 years of age (Table 4).

**Table 4.** Age-stratified multivariable associations of rural–urban locality with mood disorder and obesity prevalence by county and combined counties*^,+,#^

	Age group		
County	18–45	46–65	>65
Mood disorder			
Olmsted	1.09 (1.03–1.17)	1.26 (1.18–1.36)	1.25 (1.12–1.39)
Dodge	1.47 (1.28–1.68)	1.35 (1.14–1.59)	1.07 (0.84–1.37)
Wabasha	1.10 (0.96–1.27)	1.37 (1.17–1.61)	0.95 (0.78–1.16)
Combined counties	1.15 (1.09–1.22)	1.29 (1.21–1.36)	1.15 (1.05–1.25)
Obesity			
Olmsted	1.05 (0.96–1.16)	1.16 (1.08–1.25)	1.01 (0.91–1.13)
Dodge	1.09 (0.92–1.29)	1.16 (0.99–1.37)	1.36 (1.07–1.72)
Wabasha	1.08 (0.88–1.31)	1.12 (0.94–1.33)	0.94 (0.76–1.16)
Combined counties	1.07 (0.09–1.15)	1.15 (1.08–1.23)	1.03 (0.94–1.13)

* All analyses examined the effect of rural–urban location (reference = rural) on the condition of interest within each age group. Analyses were adjusted for sex, race/ethnicity (Non-Hispanic White vs. other), and socioeconomic status (HOUSES) quartiles.

+ Two additional multivariate models included HCU (one or more general medical examination visits, and flu vaccination, respectively) which did not modify the observed effects.

# In the analysis for combined county, the multivariable model added counties as additional adjusting factors.

## Discussion

This population-based study provides new regional data on the association of geographic locality and disparities in obesity and mood disorder over a 5-year period in a Midwestern community. In contrast with national data, our new regional results highlight heterogeneity of rural–urban disparities on health outcomes. Health disparities based on geographic location of residence on the burden of mood disorders and obesity were present, with overall prevalence higher for urban than rural residents, even after adjusting for demographics, SES, and HCU. We build on previous work by accounting for the effects of HCU and SES derived from housing features, when examining the role of rural–urban geography on disease burden. Recent attention has focused on a life-course perspective to understanding health disparities [34]. Our age-stratified analyses suggest that the strongest effects for urban locality on burden of mood disorders and obesity were seen for those in middle age (46–65 years).

Our results for prevalence of mood disorders are in contrast to prior work that found no differences based on location of residence [11,17,18]. A prior study conducted by our team [35] examined prevalence of mood disorders, along with four other health conditions, by HOUSES and race/ethnicity in a mixed rural–urban Minnesota population-based sample. That investigation found that health disparities still existed across different SES levels, with greater prevalence of mood disorder observed with higher SES among minority subjects. However, that study did not examine geographic location as a possible contributor to the burden of mood disorders.

Our results for higher obesity prevalence (although with small effect size and not consistent across counties) among urban residents are interesting, given the substantial body of past work demonstrating that rural residents have greater rates of obesity compared to their urban counterparts [3,10–14,36,37]. Another study including a regional sample [21] also observed that severity of obesity was worse among urban compared to rural residents. Collectively, these findings highlight the need to analyze and report regional data on disease burden for relevance to local communities served by the CTSAs and the need to identify subgroups of populations at risk of mood disorder and obesity in both rural and urban settings.

Although SES measures based on self-reported and aggregated measures (i.e., education and income) were accounted for in most epidemiological studies, less attention has focused on environmental factors such as neighborhood/housing contexts. For example, Wen *et al.* [15] found that the higher prevalence of obesity in rural areas was explained by individual-level (education) and economic (median household income) SES indices, but also neighborhood-level built environmental features (e.g., spatial park accessibility and walkability). Our results further indicate that urban residence effects for obesity were smaller when taking into consideration a housing-derived SES measure.

*Why is the overall prevalence of mood disorders and obesity higher for urban compared with rural residents*? Urban residents appeared to have *lower SES* as measured by HOUSES index, and generally lower HCU. However, these factors were accounted for in our analyses. Future studies could assess community and neighborhood-built environmental factors (e.g., walkability and access to parks) that may account for the observed associations with obesity risk [38,39]. Another consideration for mood disorders is that mental illness is a highly stigmatized condition [40,41] that may present barriers to diagnosis because people are less willing to discuss their symptoms at health care visits. There is some evidence that some rural communities normalize depression and other types of mental illness and have a culture of resiliency, self-reliance, and avoidance of asking for help, even when needed [42–44].

### Strengths

One strength of this investigation is the population-based study design. This study represents nearly a complete population across three counties in an area that uniquely encompasses both rural and urban communities. Many population studies have utilized age-limited data sources (e.g., Medicare). Consistent with a life-course perspective for understanding health disparities [34], our age-stratified findings add to the assessment of the effect of urban locality on burden of mood disorder and obesity prevalence. Another strength is that the prevalence of obesity and mood disorder diagnoses was based on documented provider clinical diagnosis and electronic medical record data, in contrast to reliance on self-report. The HOUSES index is an innovative, individual-level objective SES measure implementable on a large scale and is not a proxy assessment of SES drawn from aggregated measures, nor self-reported measure.

### Limitations

Our study has an inherent limitation for a cross-sectional study. In addition, our sample comprised a fairly homogeneous population of predominantly non-Hispanic Whites primarily among the rural population. Considering that the spatial domain of our study area is fairly small, it is possible that this population may have different resiliency due to unmeasured cultural or community characteristics. Thus, our results may not generalize to other geographic regions and populations. On the other hand, the homogeneity of the population allowed us to more easily disentangle the impact of SES from race/ethnicity on outcomes. Outcome measures were based on ICD codes instead of using standardized questionnaires or structured diagnostic interviews for mood disorders or body mass index (BMI) for obesity. Our study findings needed to be assessed by using different ascertainment methods. However, given the large scale of the REP study, assessing changeable longitudinal outcomes might be challenging with repeated surveys and BMI measurements over time. We did not measure a broad range of health care access (e.g., health insurance, distance from home to health care facility) in analyzing and interpreting our study results, which would help us discern whether and which health care access measures can potentially account for the observed effects. We did not assess education or income, factors that may influence prevalence of mood disorders and obesity [15,45] but HOUSES is closely correlated with, and provides unique information beyond, both SES indicators [26].

### Conclusion

In this Midwestern community, rural–urban health disparities for burden of mood disorders and obesity are independent of SES and HCU. It is important to recognize regional heterogeneity of rural–urban disparities on health outcomes.

### Implications

Our approach provides guidance to other CTSAs for assessing rural–urban disparities of disease burden within their local communities, including the need to measure and account for HCU and expanded objective measures of SES based on the social determinants of health framework. Our study results provide an opportunity and important venue for assessing the impact of rurality on health outcomes in the context of engaging and integrating special populations in clinical and translational research.

Results will be used by our community academic partnership with stakeholders from all three communities to guide development of culturally relevant and sustainable interventions focused on mental and behavioral health promotion. Community engagement platforms to increase awareness of and decrease stigma about mental health and obesity may be useful to promote care-seeking. For example, we have implemented Garden Cafes [46] in Olmsted County which could be expanded to other locations. Another specific implication of our study findings is that CTSA hubs may consider offering health promoting activities to study participants during their study visits. Special populations such as lower SES and underserved minorities might have unmet needs of health care, and thus, offering health promoting/addressing health care needs (e.g., from connection with community resources to referring to social worker) may enhance both engagement of special populations and improving health or health care access.

Across the USA, mental health ranks at or near the top of both hospital and public health community health needs assessments in both rural and urban communities [47]. Likewise, our local county health needs assessments identified mental health and obesity as top community health priorities. Our data therefore provide important baseline information for local county health departments and healthcare systems to measure the success (*impact*) of community-engaged mental and behavioral health promotion efforts over time [7].

## Supplementary material

For supplementary material accompanying this paper visit https://doi.org/10.1017/cts.2020.27. click here to view supplementary material
