# Supplementary material for: Rural–urban health disparities for mood disorders and obesity in a midwestern community
Source: J Clin Transl Sci. 2020 Mar 24;4(5):408–15. doi: 10.1017/cts.2020.27 (PMC7681122; doi:10.1017/cts.2020.27)
Supplement: Supplementary file 1 [file S2059866120000278sup001.docx]

##
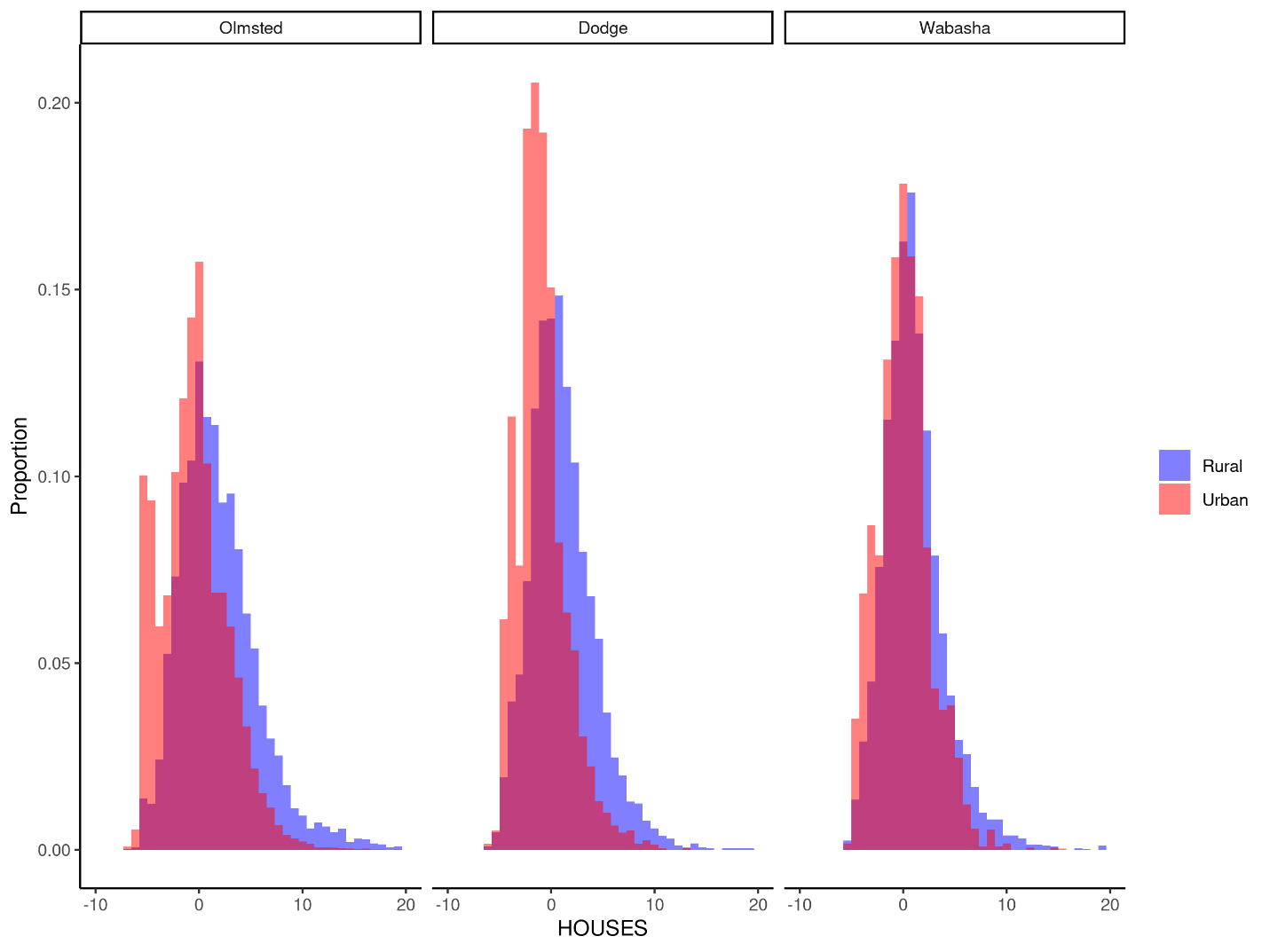
Supplementary Figure S1. *HOUSES Distribution by rural-urban status among study subjects in 3 Minnesota counties*
